# Supplementary material for: Systematic review and meta-analyses: What has the application of Mendelian randomization told us about the causal effect of adiposity on health outcomes?
Source: Wellcome Open Res. 2023 Sep 13;7:308. Originally published 2022 Dec 21. [Version 2] doi: 10.12688/wellcomeopenres.18657.2 (PMC11225060; doi:10.12688/wellcomeopenres.18657.2)
Supplement: Peer review history

# Reviewer report: Reviewer response for version 2

**DOI:** 10.21956/wellcomeopenres.22182.r83673

**Published:** 2024-07-03

**Authors:** Charlie Keown-Stoneman

**Affiliations:** 1 Applied Health Research Centre, Unity Health Toronto, Toronto, Ontario, Canada; 2 Dalla Lana School of Public Health, University of Toronto, Toronto, Ontario, Canada

This is a great paper that performed a meta-analysis and systematic review of MR studies looking at the associations between adiposity and various other health outcomes.

 I request just a few minor clarifications about the methodology. Specifically,

1. Why was the specific date, 2019-02-18 chosen as the cut-off for publication? The justification of pre-COVID-19 pandemic is understood, but why specifically that date?
2. In the data extraction section, you mention, "Where there were too few outcomes to make a category, they were grouped into an 'other' category." What was the threshold for this classification?
3. For binary outcomes results are presented as odds-ratios, for papers that reported other outcome measures (such as risk ratios or hazards-ratios) how were they transformed?

The rest of the paper seem well thought out and well referenced for the applicable methods used.

Are the rationale for, and objectives of, the Systematic Review clearly stated?

Yes

Is the statistical analysis and its interpretation appropriate?

Yes

If this is a Living Systematic Review, is the ‘living’ method appropriate and is the search schedule clearly defined and justified? (‘Living Systematic Review’ or a variation of this term should be included in the title.)

Not applicable

Are sufficient details of the methods and analysis provided to allow replication by others?

Partly

Are the conclusions drawn adequately supported by the results presented in the review?

Yes

Reviewer Expertise:

Statistics, Biostatistics, Childhood growth

I confirm that I have read this submission and believe that I have an appropriate level of expertise to confirm that it is of an acceptable scientific standard.

# Reviewer report: Reviewer response for version 2

**DOI:** 10.21956/wellcomeopenres.22182.r67014

**Published:** 2023-11-14

**Authors:** Brenda Cabrera Mendoza

**Affiliations:** 1 Department of Psychiatry, Yale School of Medicine, West Haven, Connecticut, USA

Although the manuscript improved in its revised version, the authors did not provide a clear reasoning to not updating their search, which I consider crucial. The higher volume of studies on the topic due to the COVID-19 pandemic do not seems a valid reason to limit their search to a period before this event. Actually, it makes it more important to consider such studies. I still recommend authors update their search to provide a comprehensive systematic review.

Are the rationale for, and objectives of, the Systematic Review clearly stated?

Yes

Is the statistical analysis and its interpretation appropriate?

Yes

Are sufficient details of the methods and analysis provided to allow replication by others?

Yes

Are the conclusions drawn adequately supported by the results presented in the review?

Yes

Reviewer Expertise:

Genetics; Genomics

I confirm that I have read this submission and believe that I have an appropriate level of expertise to confirm that it is of an acceptable scientific standard, however I have significant reservations, as outlined above.

# Reviewer report: Reviewer response for version 1

**DOI:** 10.21956/wellcomeopenres.20690.r57328

**Published:** 2023-06-19

**Authors:** Brenda Cabrera Mendoza

**Affiliations:** 1 Department of Psychiatry, Yale School of Medicine, West Haven, Connecticut, USA

Lee *et al.,* performed a MR meta-analysis and systematic review of the effect of adiposity on health outcomes. Overall, the manuscript is well-organized and clearly written. Also, the methods adhered to current standards. However, I have the following suggestions:

1. In the Introduction, please consider including a brief definition of MR studies. This will be helpful for readers unfamiliar with this method. 
2. It is necessary to update the search since it has been almost four years since it was performed and several relevant studies for this review have been published.
3. Since the study is focused on health outcomes, I suggest including mediRxiv among the consulted databases.
4. Indicate whether only two-sample MR analyses were included in this review or also multivariable MR studies were considered. 

Are the rationale for, and objectives of, the Systematic Review clearly stated?

Yes

Is the statistical analysis and its interpretation appropriate?

Yes

Are sufficient details of the methods and analysis provided to allow replication by others?

Yes

Are the conclusions drawn adequately supported by the results presented in the review?

Yes

Reviewer Expertise:

Genetics; Genomics

I confirm that I have read this submission and believe that I have an appropriate level of expertise to confirm that it is of an acceptable scientific standard, however I have significant reservations, as outlined above.

# Response

**Published:** 2023-09-06

**Authors:** Matthew Lee

**Affiliations:** International Agency for Research on Cancer, France

We thank the reviewer for their time in reviewing this work!

- *In the Introduction, please consider including a brief definition of MR studies. This will be helpful for readers unfamiliar with this method. *

 We have added a sentence in the first paragraph with additional references: “Briefly, MR is an epidemiological method which, exploiting the random allocation of alleles during gametogenesis, utilizes germline genetic variation (usually, single nucleotide polymorphisms *,* SNPs) as instrumental variables for modifiable exposure traits of interest ^1–3^.”

- *It is necessary to update the search since it has been almost four years since it was performed and several relevant studies for this review have been published.*

 Please see our response to comment 1 made by Reviewer 1.

- *Since the study is focused on health outcomes, I suggest including mediRxiv among the consulted databases.*

This is a valid point, as medRxiv is an important resource. However, medRxiv was not launched until June 2019. Given the scope of this systematic review and date by which we finished the searches prior to the COVID-19 pandemic (i.e., up-to February 2019), medRxiv inclusion was not within the scope of this work.

- *Indicate whether only two-sample MR analyses were included in this review or also multivariable MR studies were considered. *

This was highlighted in Extended Data 3 (i.e., the data extraction manual), but we have now made this clearer in the Methods section of the main body: *“* *All MR approaches (e.g., one-sample, two-sample, and multi-variable MR), were considered for inclusion* *.”*

# Reviewer report: Reviewer response for version 1

**DOI:** 10.21956/wellcomeopenres.20690.r53783

**Published:** 2023-01-12

**Authors:** C. Mary Schooling

**Affiliations:** 1 School of Public Health, Li Ka Shing Faculty of Medicine, The University of Hong Kong, Pokfulam, Hong Kong

This is a systematic review and meta-analysis of Mendelian randomization (MR) studies of adiposity on disease and risk factors pre-COVID, along with a survey of the available studies in that time frame. Generally, it found adiposity adversely associated with diseases or their risk factors. It is a well-conducted study adhering to current standards. Nevertheless, there are three issues.

 First, there is already a review and meta-analysis of MR studies of body mass index on different diseases, which appears to be more up-to-date and comprehensive (Larsson & Burgess 2021) ^1^. The authors need to compare their results with those of the more recent and possibly more up-to-date systematic reviews and meta-analyses, and explain the value of what appears to be a less comprehensive investigation of a similar topic. The authors need to explain why any differences in results between their work and the more recent meta-analyses have occurred. Ideally, the authors should update this systematic review and meta-analysis to include all relevant MR studies up until now. Alternatively, the authors could change their paper to be a survey of the types of MR studies that have been conducted to date on adiposity and disease outcomes and risk factors, for example more like a scoping review. Such a scoping review would however need to be up-to-date.

 Second, MR studies are open to selection bias because of the misalignment of the time of the start of exposure and of recruitment ^2^. Specifically, genetic endowment (used to instrument MR studies) starts at birth, whilst almost all the studies used were recruited in middle age or later. People who died before recruitment because of their genetically determined adiposity or died before recruitment from a disease of interest, or a competing risk of that disease, are missing. Missing people on exposure and outcome creates selection bias, which attenuates or reverses estimates ^3^. As such, some of the estimates may be misleading, particularly for late onset diseases. Ideally, the authors should consider the possibility of selection bias from selecting on survival for all outcomes considered and explain how the estimates might or might not be biased.

 Third, the key question about the role of adiposity in disease is the shape of the association. Specifically, observationally, the association of adiposity with disease may appear to be non-linear, particularly in the lower ranges ^4^. From a public health point of view, we need to know if the relation of adiposity with disease is the lower the better or if there is some optimal value. The paper does not address this key question, but at least it could be acknowledged as a limitation.

Are the rationale for, and objectives of, the Systematic Review clearly stated?

Yes

Is the statistical analysis and its interpretation appropriate?

Partly

Are sufficient details of the methods and analysis provided to allow replication by others?

Yes

Are the conclusions drawn adequately supported by the results presented in the review?

Partly

Reviewer Expertise:

Mendelian randomization; chronic disease epidemiology

I confirm that I have read this submission and believe that I have an appropriate level of expertise to confirm that it is of an acceptable scientific standard, however I have significant reservations, as outlined above.

## References

[1] : Causal role of high body mass index in multiple chronic diseases: a systematic review and meta-analysis of Mendelian randomization studies. *BMC Med* .2021;19(1) : 10.1186/s12916-021-02188-x 320 10.1186/s12916-021-02188-x 34906131 PMC8672504

[2] : Specifying a target trial prevents immortal time bias and other self-inflicted injuries in observational analyses. *J Clin Epidemiol* .2016;79: 10.1016/j.jclinepi.2016.04.014 70-75 10.1016/j.jclinepi.2016.04.014 27237061 PMC5124536

[3] : Mendelian randomization. *Nature Reviews Methods Primers* .2022;2(1) : 10.1038/s43586-021-00092-5 10.1038/s43586-021-00092-5 PMC761463537325194

[4] : Association of BMI with overall and cause-specific mortality: a population-based cohort study of 3·6 million adults in the UK. *The Lancet Diabetes & Endocrinology* .2018;6(12) : 10.1016/S2213-8587(18)30288-2 944-953 10.1016/S2213-8587(18)30288-2 PMC624999130389323

# Response

**Published:** 2023-09-06

**Authors:** Matthew Lee

**Affiliations:** International Agency for Research on Cancer, France

We thank the reviewer for their time and effort in reviewing our work, it is much appreciated and we think the paper is better for it.

- *First, there is already a review and meta-analysis of MR studies of body mass index on different diseases, which appears to be more up-to-date and comprehensive (Larsson & Burgess 2021) 1. The authors need to compare their results with those of the more recent and possibly more up-to-date systematic reviews and meta-analyses, and explain the value of what appears to be a less comprehensive investigation of a similar topic. The authors need to explain why any differences in results between their work and the more recent meta-analyses have occurred. Ideally, the authors should update this systematic review and meta-analysis to include all relevant MR studies up until now. Alternatively, the authors could change their paper to be a survey of the types of MR studies that have been conducted to date on adiposity and disease outcomes and risk factors, for example more like a scoping review. Such a scoping review would however need to be up-to-date.*

We thank the reviewer for this fair comment. By way of comparison, we found similar results in our meta-analyses compared to the review by Larsson and Burgess. For example, our meta-analysis results indicated that body mass index (BMI) increased the risk of colorectal cancer (OR: 1.18; 95% CI: 1.01–1.37), as did the meta-analysis conducted by Larsson and Burgess (OR: 1.13; 95% CI: 1.06–1.20). Where we do see inconsistent results, they typically manifest as differences in the magnitude of effect estimates and not in the direction of effect estimates. For instance, our study found a larger effect of BMI on ovarian cancer (OR: 1.39; 95% CI: 1.20–1.61) compared to the meta-analysis conducted by Larsson and Burgess (OR: 1.07; 95% CI: 0.97–1.18). Whilst both systematic reviews are valuable, there are important differences in the scope of these papers. Firstly, while Larsson and Burgess include studies published after those included in our systematic review, they focused solely on BMI and chronic diseases, whilst our study included a more comprehensive investigation of all adiposity measures with no restriction with respect to outcome. This is reflected in the larger number of articles in our review (173 vs. 47) and resultant overall number of analyses (2,214 vs. 101). Whilst Larsson and Burgess were able to perform more meta-analyses (63 in total) compared to our study (31), this was a result of the greater number of studies conducted using UK Biobank and the inclusion of *de novo* MR analyses authors conducted themselves using FinnGen, both of which featured prominently in the other review. These features highlight differences in scope and also the relative niches these two reviews occupy. Nonetheless, we wish to acknowledge previous work and the similarities and differences compared to our study and have therefore added a new paragraph to the discussion: “Results from the meta-analyses conducted here are corroborated by a recent review by Larsson and Burgess (2021) ^4^, where meta-analysis results are largely consistent. For example, our meta-analysis results indicated an effect of BMI on CRC (OR: 1.18; 95% CI: 1.01–1.37) and venous thromboembolism (OR: 1.58; 95% CI: 1.33–1.87), estimates of which were similar to those provided by Larsson and Burgess for the same phenotypes (i.e., OR: 1.13; 95% CI: 1.06–1.20 for CRC; and OR: 1.49; 95% CI: 1.33–1.66 for venous thromboembolism). Whilst the direction of effect estimates were largely consistent across both meta-analyses, where we do see inconsistent results, they typically manifest as differences in the magnitude of effect estimates. For instance, our study found a larger effect of BMI on ovarian cancer (OR: 1.39; 95% CI: 1.20–1.61) compared to the review by Larsson and Burgess (OR: 1.07; 95% CI: 0.97–1.18). Such differences in magnitude may reflect more reliable estimates obtained from studies with larger case numbers as used in their review (cases for ovarian cancer = 27,420) compared to our meta-analysis (cases for ovarian cancer = 5,400), due to the predominance of studies using UK Biobank included in their meta-analysis. There are also important differences in the scope of these papers. Firstly, whilst the review published by Larsson and Burgess specifically focused on BMI and a handful of chronic disease groups, many of which overlapped with the outcomes included in our work, our study was broader, encompassing all adiposity measures, with no restriction on outcome. Similarly, whilst the date by which the searches were completed in our systematic review was earlier, we included a larger number of articles (173 vs. 47), extracted data on more MR studies (2,214 vs. 101) and did not exclude studies that had investigated the same outcome using the same population. Lastly, we also provided a narrative synthesis of data extracted from all 2,144 MR studies not included in the meta-analyses.” Regarding the reviewer’s second point about updating the systematic review, we agree, and acknowledge in the final paragraph of our introduction, that an update to this work would be valuable. However, this update is not within the scope of this current review given our aim to target the pandemic window of publication before there was a systemic shift in publication characteristics – especially for BMI. In addition to this, we are relatively confident that the inclusion of an update would likely lead to the similar conclusions – not justifying a lack of update (given the focus of our review), but noting that the publication our specific review would not substantively alter the current evidence base, but work with it. As an indication, we performed a preliminary search of EMBASE and MEDLINE from February 2019 – July 2023 and identified 5,106 articles (after de-duplication). If we assumed a similar inclusion rate as we published here (i.e., ~2.6%), we would expect an additional ~105 articles to be included for further data extraction in efforts to update the current review. Updating the review would require resources that are beyond the remit of the existing review project.

- *Second, MR studies are open to selection bias because of the misalignment of the time of the start of exposure and of recruitment 2. Specifically, genetic endowment (used to instrument MR studies) starts at birth, whilst almost all the studies used were recruited in middle age or later. People who died before recruitment because of their genetically determined adiposity or died before recruitment from a disease of interest, or a competing risk of that disease, are missing. Missing people on exposure and outcome creates selection bias, which attenuates or reverses estimates 3. As such, some of the estimates may be misleading, particularly for late onset diseases. Ideally, the authors should consider the possibility of selection bias from selecting on survival for all outcomes considered and explain how the estimates might or might not be biased.*

This is an important point and we have now added a statement to this end in the discussion: “In addition, the potential for selection bias, particularly in studies of later life diseases such as Alzheimer’s *disease*, may lead to attenuated or reversed estimates due to selection bias ^5^. This may arise when genetic variants associated with the exposure (in this case BMI) influence study participation and will be most prominent for exposures that may cause diseases that prevent the study of later life diseases because of mortality or loss to follow-up ^5^. Whilst this may be present within the current evidence synthesis, particularly for those disease and health outcomes occurring in middle-to-late life, it is difficult to ascertain and quantify the impact that this may have on the current estimates. We advocate that authors undertaking MR analyses on such outcomes consider this when conducting and interpreting their estimates.”

- *Third, the key question about the role of adiposity in disease is the shape of the association. Specifically, observationally, the association of adiposity with disease may appear to be non-linear, particularly in the lower ranges4. From a public health point of view, we need to know if the relation of adiposity with disease is the lower the better or if there is some optimal value. The paper does not address this key question, but at least it could be acknowledged as a limitation.*

This is a good point and we agree that we should acknowledge this important issue. As such, we have added the following text to the discussion: “It is also important to note that a key assumption of most MR analyses is that there is a linear association between the exposure on the outcome and between the genetic instrument and exposure. Few studies included within our research acknowledged this assumption *nor *the potential bias non-linear effects may have on estimated effects. Investigations of non-linear effects were primarily performed in methodological studies in which an adiposity measure was used as an exemplar ^6,7^. Whilst there may be causal non-linear effects between adiposity and some diseases or health outcomes, this is currently difficult to estimate with MR methodologies ^8–10^ and, in an observational context, non-linear relationships may instead reflect bias (e.g., due to reverse causation or confounding). For example, in the context of BMI and mortality, the presence of those who lose weight due to co-morbidities that increase the risk of mortality in the study sample may indicate that a lower BMI increases the risk of mortality). Until more concrete non-linear methodologies are developed, this will remain a key limitation of providing unbiased non-linear effect estimates of the causal role of adiposity on health outcomes.”
